# Dehydroepiandrosterone decreases the age-related decline of the in vitro fertilization outcome in women younger than 40 years old

**DOI:** 10.1186/s12958-015-0014-3

**Published:** 2015-03-09

**Authors:** Massimo Tartagni, Maria V Cicinelli, Domenico Baldini, Mario V Tartagni, Hala Alrasheed, Maria A DeSalvia, Giuseppe Loverro, Monica Montagnani

**Affiliations:** Department of Biomedical Sciences and Human Oncology, School of Medicine, University of Bari “Aldo Moro”, Bari, Italy; Centro di Fecondazione Medicalmente Assistita MoMò Fertilife, Bisceglie, Italy

**Keywords:** Dehydroepiandrosterone (DHEA), Pregnancy rate, IVF parameters

## Abstract

**Background:**

With infertility populations rapidly aging, treatments improving pregnancy chances assume increasing clinical importance. Dehydroepiandrosterone (DHEA) has been reported to improve pregnancy rates and lower miscarriage rates in women with diminished ovarian function. This study was planned to evaluate whether pretreatment with DHEA may improve *in vitro* fertilization (IVF) parameters and pregnancy outcomes in infertile women with advanced reproductive age and normal ovarian reserve.

**Methods:**

In this double-blind, randomized, placebo-controlled study, 109 infertile patients aging 36–40 years old were selected to undergo the long protocol IVF. Eight weeks before starting the IVF cycle and during treatment, patients in Group 1 received 75 mg of DHEA once a day; patients in control group (Group 2) received placebo. The primary endpoint of the study was number of clinical pregnancy, live birth and miscarriage rates; secondary endpoint was modification of standard IVF parameters, including stimulation duration (days of rhFSH administration), E2 on HCG-day, endometrial thickness, number of retrieved oocytes, metaphase II oocytes, number of transferred embryos and score of leading embryos transferred.

**Results:**

Patients in the DHEA group had a significantly higher live birth rate compared with controls (P < 0.05). Conversely, miscarriage rate was higher for patients in the control group (P < 0.05).

**Conclusions:**

DHEA supplementation may significantly improve IVF outcomes in infertile women with advanced reproductive age and normal ovarian reserve.

## Background

As a consequence of multiple inherited and acquired factors related to life style, such as exposure to oxidative stress and/or endocrine disruptors, the ability to conceive naturally declines over time [1]. The progressive decline in women’s fertility potential together with the late age of motherhood is constantly increasing the number of patients consulting infertility specialists. Among a wide range of strategies proposed to patients close to 40 years in order to overcome their infertility and improve the live birth rate, increasing attention has been aimed at treatments involving dehydroepiandrosterone (DHEA), an endogenous adrenal steroid from ovarian theca cells and adrenal cortex. DHEA is an essential pro-hormone in ovarian follicular steroidogenesis and it is used as an anabolic steroid by the athletes [2].

Because of its potential to slow down and reverse the effects of aging, DHEA has been proposed as novel treatment to improve the fertility rate in women with diminished ovarian reserve (DOR) responding poorly to gonadotrophin stimulation, and in women of advanced age [3]. Benefits of DHEA supplementation have been reported since the beginning of this decade [4-8], and recent studies further emphasize the association between DHEA supplementation and the rate of spontaneous and treatment-induced pregnancies in patients with very high follicle-stimulating hormone (FSH) or very low anti-mullerian hormone (AMH) levels [9-11].

In women with a significant DOR, DHEA treatment has been related to amelioration of outcome parameters of *in vitro* fertilization (IVF) such as peak estradiol level, numbers and quality of embryos, reduced miscarriage rates [12-14]. These findings suggest that beneficial effects of DHEA supplementation might be even greater in patients with normal ovarian reserve undergoing IVF treatment [15]. Nevertheless, in the absence of randomized studies, DHEA cannot be recommended as a routine protocol in advanced reproductive age.

In several countries, DHEA is sold over the counter as food supplement; but even in those countries where it is only available as a prescription drug, DHEA may be used outside the scope of its approved indications. The need of randomized studies is even more compelling when considering that DHEA is becoming popular as self-medication, but that analysis of commercially available DHEA products (not derived from human sources) found that DHEA content ranged from 0 to 150% of the labelled amount [16,17].

Based on these observations, this study was undertaken to assess the potential benefits of DHEA supplementation in women with advanced reproductive age, but normal ovarian reserve, undergoing IVF program.

## Methods

All procedures were in accordance with the Helsinki Declaration on Human Experimentation and approved by the local Ethic Committee. A randomized, prospective, placebo-controlled trial was carried out to evaluate the effects of DHEA administration in patients with advanced reproductive age undergoing their first IVF cycle. For this study, 109 patients with a diagnosis of infertility and undergoing their first IVF cycle were consecutively recruited between January 2010 and October 2012 among those coded in the anonymous research database of our academically affiliated private infertility center. All patients enrolled had unsuccessfully sought to became pregnant for more than 3 years and had failed at least 3 intrauterine inseminations (*IUI*).

Each eligible participant received a detailed explanation on the importance of ovarian response in IVF cycles, on possible beneficial and side effects of DHEA supplementation, and on the significance of a randomized controlled trial investigating the effect of DHEA in women on advanced reproductive age. Those subjects consenting to be enrolled and fulfilling the inclusion criteria were asked to sign a written informed consent.

### Recruitment and inclusion/exclusion criteria

Participants’age ranged from 36 to 40 years. All the subjects were regularly menstruating (menstrual cycle length: 24 – 34 days), had normal BMI values and normal ovarian reserve, assessed as basal FSH (cycle day 3), AMH, or inhibin B levels in the normal range (Local standard: FSH < 10 IU/L; AMH 2.0-6.80 ng/ml; inhibin B > 45 pg/ml). Basal hormone values and antral follicle count in each ovary were measured in the cycle preceding IVF. A routine infertility evaluation, including semen analysis and spermiocolture was normal in all patients’ sexual partners. Moreover, no participant had a history of ovarian or pelvic surgery; none of them reported a diagnosis of endocrine or metabolic disorders, including polycystic ovarian disease (PCOS). Finally, none of the enrolled patients had taken oral contraceptives or other hormone therapy within the last 6 months. Patients were excluded if their age was over 40, if they had received DHEA at any time before enrollment, if they had failed a previous IVF cycle and if a history of cardiovascular, thromboembolic, hepatic or renal disease was reported.

### Treatment protocol

On the basis of a computer-generated randomization sequence, patients recruited were assigned to 2 treatment groups. In Group 1 (n = 53) patients were administered with DHEA (75 mg/die, orally) 8 weeks before starting the cycle of ovulation induction. Patients of Group 2 (n = 56) received placebo during the same period. Both DHEA and placebo were administrated throughout the whole period of ovarian stimulation up to β-HCG test. DHEA dispensed in this study was obtained by a single pharmacy.

Eight weeks after DHEA pretreatment, IVF procedure was initiated according to our standard long-stimulation protocol [15]. Briefly, pituitary suppression was achieved by administration of triptorelin acetate (Decapeptyl®, Ferring, Germany) (1 mg/die, subcutaneously) in the mid-luteal phase of the cycle preceding the IVF procedure. When down-regulation was achieved (indicated by estradiol (E_2_) concentration < 50 pg/ml and absence of follicles > 10 mm diameter), recombinant human FSH (Gonal F®, Merck-Serono SA, Aubunne, Switzerland) (300 IU/die, subcutaneously) was administered. Starting from day 5, rhFSH was administered according to individual ovarian response, as assessed by serum levels of E_2_ and sequential transvaginal ultrasonography; when the leading follicle(s) reached a 18 mm diameter, recombinant human CG (Ovidrel®, Merk-Serono SA, Bari, Italy) (500 μg/ subcutaneously) was injected. Oocytes aspiration was performed 36 hours later.

Subsequently, embryos obtained were graded according to published criteria [15]; embryos of grade 1 or 2 were considered of high quality. On day 3 after oocyte recovery, up to 3 best-quality embryos were transferred. Vaginal micronized progesterone (600 mg/day given at 8-h intervals) was started on the day following oocyte aspiration and continued for an additional 9 weeks to support luteal phase in case of pregnancy. Pregnancy was diagnosed by measuring increasing serum levels of β-hCG 12 days after hCG administration. Clinical pregnancy was established if a fetal heart beat was observed by transvaginal ultrasound.

### Study end-points

The number of clinical pregnancies was considered as the primary end-point for this study, with the live birth rates and the miscarriage rates assumed as correlated parameters of the primary end-points. Secondary end-points were standard IVF parameters, such as stimulation duration (days of rhFSH treatment), E_2_ levels on hCG-day, endometrial thickness, number of retrieved oocytes, number of metaphase II oocytes, number of embryos transferred and score of leading embryos transferred.

### Statistical analysis

Data were analyzed using Statistica software from StatSoft (Tulsa, Oklahoma, USA). Student’s *t*-test and Fisher’s exact test were used, as appropriate. Results are expressed as mean ± standard deviation (SD), unless otherwise specified. A P value < 0.05 was considered to indicate statistical significance.

## Results

The study was carried out from January 2010 to October 2012. During this time-frame, 115 patients who met the criteria for the study were identified; of these subjects, 4 were exposed to DHEA treatment before they came to our observation and 2 did not provide their consent to participate. Therefore, a total of 109 women were enrolled and randomly assigned to Group 1 (DHEA, n = 53) or Group 2 (Control, n = 56). All patients in both groups completed the study.

Table 1 summarizes patient characteristics. Mean age was comparable in patients of Group 1 (37.1 ± 2.2) and Group 2 (37.4 ± 2.9). Similarly, BMI values did not differ in patients from Group 1 (22.1 ± 1.6) with respect to Group 2 (22.5 ± 1.4). Mean basal values of serum FSH, AMH, and inhibin B did not significantly differ between the two groups, as well as antral follicle count on day 3 of the cycle preceding the IVF procedure. This supports the validity of randomization process.

**Table 1:** **Baseline characteristics of patients in Group 1 and Group 2**

**Variables**	**Group 1**	**Group 2**
Number (n^o^) of patients	53	56
Age (years)	37.1 ± 2.2	37.4 ± 2.9
BMI (kg/m^2^)	22.1 ± 1.6	22.5 ± 1.4
FSH (IU/l)	6.5 ± 1.3	6.38 ± 1.3
AMH (ng/ml)	5.7 ± 1.2	5.5 ± 1.4
Inhibitin B (pg/ml)	49 ± 3.2	48.5 ± 3.8
Antral follicle count (n^o^)	12.7 ± 3	13.2 ± 2.5

Values are mean ± SD. No significant difference (p > 0.05) was observed between groups for all tested parameters.

Significant differences in Group 1 with respect to Group 2 were observed for several parameters, including duration of ovary stimulation (hCG-day) (11 ± 0.8 *vs.* 13.1 ± 1.1; P < 0.05), serum levels of E_2_ on hCG-day (3478 ± 621 *vs.* 2051 ± 887 pg/ml; P < 0.01), and endometrial thickness (10.4 ± 0.9 mm *vs.* 9.1 ± 1.8 mm; P < 0.0001) (Table 2). Moreover, although statistically significant differences were not reached, the number of retrieved oocytes found in patients undertaking DHEA was more elevated than in those taking placebo (8.9 ± 1.8 *vs.* 8.2 ± 2.2; P > 0.07). Concomitantly, metaphase II oocytes (6.1 ± 1.3 *vs.* 5.8 ± 1.1; P > 0.11), number of embryos transferred (2.5 ± 0.6 *vs.* 2.3 ± 0.7; P > 0.13) and number of clinical pregnancies (22 *vs.* 18; P > 0.15) were all showing trends of increase in patients exposed to DHEA with respect to patients administered with placebo (Table 2).

**Table 2:** **Outcomes of patients in Group 1 and Group 2**

**Variables**	**Group 1**	**Group 2**	**P value**
Number (n^o^) of patients	53	56	
hCG day	11 ± 0.8	13.1 ± 1.1	<0.05
E_2_^a^ on hCG day (pg/ml)	3478 ± 621	2051 ± 887	<0.01
Endometrial thickness (mm)	10.4 ± 0.9	9.1 ± 1.8	<0.0001
N^o^ of retrieved oocytes^b^	8.9 ± 1.8	8.2 ± 2.2	n.s. (0.07)
N^o^ of methaphase II oocytes^b^	6.1 ± 1.3	5.8 ± 1.1	n.s. (0.11)
N^o^ of embryos transferred^b^	2.5 ± 0.6	2.3 ± 0.7	n.s. (0.13)
High quality embryos replaced^b^ (%)	77.3	48.2	<0.01
Clinical pregnancy (n°)	22	18	n. s. (0.15)
Live birth rate	22	13	<0.05
Miscarriage rate	0	5	<0.05

Values are mean ± SD unless otherwise specified. ^a^Estradiol values. ^b^Values are relative to the n^o^ of patients with oocyte retrieval. n. s. = not significant.

Of the 22 pregnant patients in Group 1, 21 delivered a live singleton infant and 1 patient had a twin birth; no miscarriage was reported. Conversely, among the patients of control Group 2, 5 out of 18 pregnancies ended in an miscarriage within 9-week gestation and 13 delivered a live singleton infant. Live birth rate was significantly higher in Group 1 than in Group 2 (22 *vs.* 13; P < 0.05); conversely, miscarriage rate was higher in control Group 2 than in Group 1 (5 *vs.* 0; P < 0.05) (Table 2). The causes of the 5 miscarriages remain unclear: 4 of them occurred before the 7-week of gestation, and no further investigation was requested and carried out to elucidate the reasons. In one miscarriage, occurring in a 38-years old woman at the 9-week of pregnancy, the cytogenetic assay was showing the presence of a trisomy 21. However, no direct cause-relationship between the genetic abnormality and the miscarriage could be established.

## Discussion

Age is one significant factor influencing a woman’s ability to conceive. In Western Countries, social trends have progressively deferred childbearing, with the result that an increasing number of women are experiencing age-related infertility and pregnancy loss. The fecundity of women decreases gradually but significantly beginning the age of 32 years, and more rapidly after age 37 years. Education and enhanced awareness of the effects of aging on fertility are essential factors when counseling patients who seek pregnancy. Based on the anticipated age-related decline in fertility, the increased incidence of disorders that impair fertility, and the higher risk of pregnancy loss, the American College of Obstetricians and Gynecologists Committee has recently recommended that women older than 35 years should receive an expedited evaluation and undergo treatment after 6 months of failed attempts to conceive or, if clinically indicated, even earlier [18].

DHEA has been proposed among several disparate strategies of investigation and management to patients with diminished ovarian reserve (DOR) in order to overcome their infertility and improve the live birth rate. However, to the best of our knowledge, this is the first randomized, placebo-controlled study evaluating the potential beneficial effects of DHEA administration in women with infertility and advanced reproductive age, but with still normal ovarian reserve.

The first study to suggest therapeutic benefits from DHEA supplementation in women with DOR was carried out by Casson *and colleagues* [13]. Subsequently, and more recently, other studies have reported an improved ovarian function among patients with poor ovarian response after DHEA supplementation [4-6]. Mamas *and* Mamas [8,9] published promising findings related to DHEA administration in patients with ovarian failure. In a randomized prospective study, Wiser *et al.* [19] reported a higher pregnancy and birth rates among poor responder patients treated with DHEA, before and during IVF treatment, compared with control group. Despite promising results, a recent meta-analysis investigating the efficacy of DHEA as adjuvant therapy in patients with DOR warns about the limited evidence available, and urges the need of randomised controlled trials together with further knowledge about DHEA mechanisms of action to support the use of DHEA in standard practice for poor-responders [20].

With respect to the mechanism of action of DHEA on the ovary, much at present remains speculative: for decades androgens have been considered detrimental to follicle maturation [21]. More recently, some evidence suggests that androgens may be essential to normal folliculogenesis and female fertility [10,22,23]. In mice these effects appear granulose cell-specific [24]. It has been postulated that DHEA can improve steroidogenesis since it serves as a precursor for the production of androstenedione, testosterone and, consequently, estrogen within the ovary [10,22,23]. During ovarian induction with exogenous gonadotropins, DHEA is the prohormone of the follicular fluid testosterone [25]. In the study carried out by Casson *et al.* in post-menopausal women, the Authors evaluated whether DHEA treatment might influence the steroidogenic status, rather than the follicular development [26]. These authors reported a transient increase in insulin-like factor 1 (IGF-1) in patients undergoing exogenous gonadotropin ovulation induction after pre-treatment with DHEA. Afterwards they hypothesized that the beneficial effect of DHEA may have been mediated by increase in IGF-1.

In our study we use long protocol for induction of ovulation. Some studies have suggested that women who have been down-regulated with Gn-RH agonist an then stimulated with rh-FSH alone may experience low LH concentration that compromise the parameters of IVF treatment [27]. Current notion in folliculogenesis suggests that LH plays an essential role on follicular maturation [28]. Androgens are produced by the theca cells in response to LH. Lossl *et al.* demonstrated that increased intrafollicular androgen concentration in the early follicular phase resulted in an increased embryos quality [29]. As previously mentioned, DHEA is the precursor to androgens [28] and a prohormone for up 48% of follicular fluid testosterone [25]. Our results are consistent with these concepts, and support the idea that beneficial effect of DHEA may be linked to a more appropriate intrafollicular androgen concentration. In other words, DHEA could improve the ovarian environment in which follicular maturation takes place, and consequently ameliorate the oocytes and embryos quality. A variety of side effects, including hair loss, acne, hirsutism and voice deepening have been reported in women undergoing DHEA treatment [30], but they are negligible at the therapeutic dose of 75 mg/day employed in this study. Indeed, no androgenic effect occurred among our patients.

Along with increased pregnancy rate, another important finding of our study is the significantly lower miscarriage rate in Group 1, despite comparable age of patients in both groups (≥36 years). Aneuploidy rate increases with age [31,32], and age 35 is generally considered the cut off, when invasive prenatal genetic screening becomes indicated [33]. Since a majority of miscarriages are believed to be consequence of aneuploidy [34], it is possible to hypothesize that a decreased aneuploidy rate should be translated into decreased spontaneous pregnancy loss. On this respect, Gleicher *et al.* were able to demonstrate that DHEA supplementation was associated with significantly reduced aneuploidy in women with DOR [7], and greatest reductions were observed with short DHEA supplementation of up to 12 weeks [11]. It is unlikely that this effect may be restricted to infertile women with DOR, as in our study a reduced miscarriage rate was also observed in women without DOR administered with DHEA, with respect to patients undergoing placebo treatment. Unfortunately, the causes of the 5 miscarriages observed in patients taking placebo were not further explored: therefore, data currently available are insufficient to establish whether DHEA supplementation may concur to reduce embryo aneuploidy. Nevertheless, our data seem in line with the hypothesis that DHEA supplementation may confer additional protection against spontaneous miscarriage, and suggest that this is a potential beneficial effect also in women older than 35 years without DOR.

## Conclusions

Overall, our results suggest that pretreatment with DHEA is able to improve fertility in patients with advanced reproductive age seeking pregnancy. Although additional larger studies using different DHEA protocols are required to support our present findings, this is nonetheless the first randomized, placebo-controlled study suggesting the potential beneficial effects of DHEA administration in women with infertility and advanced reproductive age, but with still normal ovarian reserve.
